# Risk of Second Primary Malignancies in Colon Cancer Patients Treated With Colectomy

**DOI:** 10.3389/fonc.2020.01154

**Published:** 2020-07-16

**Authors:** Bo Zhang, Kaibo Guo, Xueer Zheng, Leitao Sun, Minhe Shen, Shanming Ruan

**Affiliations:** ^1^Cancer Hospital of the University of Chinese Academy of Sciences (Zhejiang Cancer Hospital), Hangzhou, China; ^2^Institute of Cancer and Basic Medicine, Chinese Academy of Sciences, Hangzhou, China; ^3^The First Clinical Medical College of Zhejiang Chinese Medical University, Hangzhou, China; ^4^Department of Medical Oncology, The First Affiliated Hospital of Zhejiang Chinese Medical University, Hangzhou, China

**Keywords:** second primary malignancy, colon cancer, prostate cancer, competing risk model, nomogram

## Abstract

**Background:** Second primary malignancy (SPM) attracts a growing attention. However, the clinical features of colon cancer (CC) survivors with SPMs are not clear and could help guide clinicians to develop a better surveillance strategy.

**Methods:** We reviewed 56,930 CC survivors treated with colectomy from the Surveillance, Epidemiology, and End Results (SEER) database during 1998–2011. Competing risk models and nomograms were conducted for predicting the risk of occurring SPMs. The clinical utility of the models was measured by decision curve analysis (DCA) using net benefit approaches.

**Results:** Five thousand thirteen (17.1%) of male patients developed SPMs and sites of SPMs included prostate (32.2%), lung and bronchus (11.6%), urinary bladder and kidney (10.8%), colon (10.0%), and melanoma of the skin (3.9%), while 3,592 (13.0%) of female patients occurred SPMs and sites of SPMs involved breast (25.8%), lung and bronchus (13.6%), colon (11.6%), uterus (8.2%), urinary bladder, and kidney (5.6%). Survivors with a second carcinoma of lung and bronchus showed the worst prognosis. Older age increased the risk of SPMs in both male (Subdistribution hazard ratio =2.85 [95% confidence interval = 2.53–3.21]) and female (1.80 [1.59–2.04]) survivors, especially for the risk of a second prostate carcinoma in male (5.33 [4.03–7.03]). Compared with white race, black male survivors remained at higher risk to develop the second prostate carcinoma (1.98 [1.74–2.26]). Competing-risk nomograms for CC survivors were established to help clinicians predict the probabilities of overall SPMs and prostate carcinoma. Validation of nomograms showed good discrimination and accuracy, and DCAs revealed the clinical effectiveness.

**Conclusions:** We profiled the clinical characteristics of a large population-based cohort of CC survivors with SPMs. These features may improve future follow-up management, especially for the surveillance of second prostate cancer in men and second breast cancer in women.

## Introduction

Colon cancer (CC) is the most commonly diagnosed digestive malignancy in the world (1, 2), and in 2020, it is estimated that it would be 104,610 incident cases in the United States (3). With the development of the early diagnosis, effective treatments and cancer surveillance, there has been seen improved in 5-year survival from 49.8 to 66.2% (4), resulting in a continuously increasing number of CC survivors. Unfortunately, it was reported that more than 8% of these survivors were facing the probability of progressing a second primary malignancy (SPM) (5) and SPM becomes one of the main causes of death for those patients (6). Hence, there are a growing number of studies exploring the risk factors of SPM in different cancer survivors such as breast (7), lung (8) as well as colorectal cancer (9, 10). However, these studies used logistic regressions or Cox proportional hazard regressions to analyze SPM-related factors, which ignored the death as a competing event for occurring PSMs. They also did not take into account the heterogeneity of SPM risk between colon and rectum, as well as male and female. Additionally, probabilities of SPMs sites and their survivals were less known and the effective nomogram for prediction was not conducted (11, 12).

The aim of the present study was to comprehensively characterize male and female CC patients suffering from SPMs, and to find out the survivals and incidence rates of top 5 SPMs sites, using two large population-based cohorts (SPM cohort and only one primary malignancy (OOPM) cohort) from the Surveillance, Epidemiology, and End Results (SEER) database. We first exhibited the differences of demographic variables and tumor characteristics between these two cohorts, and calculated the survival and cumulative incidence of top 5 SPMs sites. Then, the significant unbiased factors were demonstrated to be linked with the risk of SPMs by considering the death as a competing event. Finally, we intuitively predicted the SPM probabilities of initial CC patients and identified the survivors at high risk of SPMs by constructing competing-risk nomograms.

## Methods

### Data Sources and Population Selection

We extracted the data from the SEER 18 registry database by using SEER^*^Stat 8.3.6 software (http://seer.cancer.gov/seerstat/). Up to now, the SEER database has collected and published cancer incidence and survival data covering ~34.6% of the U.S. population. Cases of colon cancer were identified by “International Classification of Diseases for Oncology, 3rd Edition (ICD-O-3) Hist/behav, malignant.” Patients aged 20–79 years, who were initially diagnosed as colon cancer with stage I–III between January 1998 and December 2011, and underwent colectomy were included in this study, ensuring long-term follow-up of at least 5 years to exist the risk of developing SPMs. The two key variables “sequence number” and “total number of *in situ*/malignant tumors for patient” of SEER database were used to determine the status of SPM. Cases that were diagnosed as synchronous cancers occurring SPM within 2 months after initial diagnosis, were subsequently excluded. In addition, all important covariates of these cases were complete without missing values. In the next step, we divided the involved CC patients into two groups: SPM cohort and the OOPM cohort. The flowchart of cases selection was shown in Supplementary Figure 1.

### Outcome and Variable Declaration

The occurrence of a SPM after the initial primary cancer diagnosis in male and female CC survivors was studied, respectively. Overall survival (OS) was referred to the time from the initial cancer diagnosis to death, while cumulative incidence of SPM was calculated regarding the death as a competing event. For demographic characteristics, we enrolled age at initial diagnosis (18–49, 50–64, 65–79 years), race (white, black, other) and marital status (married, unmarried). Tumor covariates involved initial diagnosis site (right colon: C18.2-Ascending colon, C18.3-Hepatic flexure of colon, C18.4-Transverse colon; left colon: C18.5-Splenic flexure of colon, C18.6-Descending colon, C18.7-Sigmoid colon), tumor size (<3, 3–5, >5 cm), number of lymph nodes examined (<12, ≥12), histology (adenocarcinoma, other), tumor grade (I–II: well-differentiated or moderately-differentiated; III–IV: poorly-differentiated or undifferentiated), AJCC 6th stage (I, II, III). Treatment related variables included chemotherapy (No/Unknown, Yes), and surgery (partial colectomy, subtotal or total colectomy). Other covariates included length of follow-up (5–10, 10–15, 15–19 years), status (alive, dead) and cause of death (first primary cancer, multiple malignancies, noncancer cause).

### The Fine-Gray Proportional Subdistribution Hazards Modeling

The death of patients acted as a competing event of developing a second tumor and using Cox proportional hazards model would overestimate the incidence rate of the outcome with the passage of time (13). Thus, we used the Fine and Gray proportional subdistribution hazards model to evaluate the unbiased risks of developing a SPM, which could account for the competing event (14). Risk factors of total SPMs and top 5 SPMs for male and female CC survivors were analyzed, respectively. Candidate variables involved age at initial diagnosis, race, marital status, initial diagnosed site, tumor size, number of lymph nodes examined, histology, tumor grade, AJCC 6th stage, chemotherapy, and surgery. Multivariable competing risk models were conducted to find out the significant risk factors.

### Competing-Risk Nomogram Construction and Evaluation

In order to help the clinicians to predict the individual SPM probability of male and female CC survivors, we established nomograms on the basis of the multivariate competing risk model. Next, we identified low-, medium-, and high-risk survivors by calculating the quantiles of total points and compared the difference of the SPM incidence among these subgroups. Validation of nomogram (15) was performed by calculating the concordance index (C-index) and plotting calibration curves by a bootstrapping method with 1,000 resamples. The C-index was used to quantify the discriminatory power of the model and the calibration plots were used to evaluate the accuracy of the nomogram. Furthermore, decision curve analysis (DCA) (16) which could calculate the net benefits at each risk threshold probability, was conducted to show the clinical effectiveness of the nomogram model. As far as we know, there was no nomogram with other model. Thus, we compared the nomograms with the models consisted of demographic characteristics (age, race, marital status) or tumor covariates (tumor size, tumor site, grade, stage, etc).

### Statistical Analysis

All the data were statistically analyzed in R software (version 3.6.1, https://www.r-proje ct.org/). The Fine and Gray proportional subdistribution hazards model, competing-risk nomogram, C-index, calibration curves, Kaplan-Meier curves, cumulative incidence functions and DCA were conducted by using R 3.6.1 with relevant packages and functions, such as survival, cmprsk and stdca (https://www.mskcc.org/departments/epidemiology-biostatistics/health-outcomes/tutorial-r). A two-tailed value of *P* < 0.05 was considered statistically significant.

## Results

### Patient Enrollment and Characteristics

A total of 56,930 survivors initially diagnosed with CC were included in this study; 5,013 (17.1%) of male patients and 3,592 (13.0%) of female patients developed SPMs occurring 2 months and more after the initial diagnosis. The follow-up time of over 50% patients was more than 10 years. Moreover, we found that prostate, lung and bronchus, urinary bladder and kidney, colon, and melanoma of the skin were the five most common sites of SPMs for male CC survivors, while breast, lung and bronchus, colon, uterus, urinary bladder and kidney were top 5 SPMs sites in female CC patients. The characteristics of CC survivors stratified by gender were exhibited in Tables 1, 2, respectively. Specifically, about 60% of patients in SPM cohort were aged 65–79 years and survivors with grade I-II accounted for approximately 80%. Married black male survivors and white female survivors were linked to the increased risk of a SPM. Moreover, right colon, or tumor size >5 cm, or number of lymph nodes examined <12, or stage I in all patients with CC showed the higher SPM incidence. Of these patients with SPMs, about 30 and 50% underwent chemotherapy and partial colectomy, respectively. The various causes of death in involved survivors were also displayed in Tables 1, 2. In total, 8,261 male CC patients (28.2%) and 6,720 female CC patients (24.31%) were dead, and the proportions of survivors with SPMs for causes of death, including first primary cancer, multiple malignancies and noncancer cause, were about 25, 45, and 30%, respectively.

**Table 1:** Clinicopathological variables of male CC survivors with stratified events.

**Risk factors**				**Top 5 SPM in male**, ***n*** **(%)**				
	**Overall, *n* (%)**	**OOPM cohort, *n* (%)**	**SPM cohort, *n* (%)**	**Prostate**	**Lung and Bronchus**	**Urinary Bladder and Kidney**	**Colon**	**Melanoma of the Skin**
	**(29,290)**	**(24,277)**	**(5,013)**	**(1,613)**	**(580)**	**(540)**	**(499)**	**(195)**
**Age at initial diagnosis, years**								
18–49	3,757 (12.83)	3,450 (14.21)	307 (6.12)	53 (3.29)	19 (3.28)	18 (3.33)	58 (11.62)	13 (6.67)
50–64	11,906 (40.65)	10,099 (41.60)	1,807 (36.05)	632 (39.18)	179 (30.86)	187 (34.63)	162 (32.46)	76 (38.97)
65–79	13,627 (46.52)	10,728 (44.19)	2,899 (57.83)	928 (57.53)	382 (65.86)	335 (62.04)	279 (55.91)	106 (54.36)
**Race**								
White	23,570 (80.47)	19,493 (80.29)	4,077 (81.33)	1,241 (76.94)	484 (83.45)	469 (86.85)	389 (77.96)	192 (98.46)
Black	2,991 (10.21)	2,425 (9.99)	566 (11.29)	286 (17.73)	44 (7.59)	37 (6.85)	55 (11.02)	2 (1.03)
Other	2,729 (9.32)	2,359 (9.72)	370 (7.38)	86 (5.33)	52 (8.97)	34 (6.30)	55 (11.02)	1 (0.51)
**Marital status**								
Married	21,853 (74.61)	18,011 (74.19)	3,842 (76.64)	1,272 (78.86)	434 (74.83)	428 (79.26)	378 (75.75)	151 (77.44)
Unmarried	7,437 (25.39)	6,266 (25.81)	1,171 (23.36)	341 (21.14)	146 (25.17)	112 (20.74)	121 (24.25)	44 (22.56)
**Initial diagnosed site**								
Right colon	12,515 (42.73)	10,293 (42.40)	2,222 (44.32)	768 (47.61)	250 (43.10)	256 (47.41)	186 (37.27)	92 (47.18)
Left colon	16,775 (57.27)	13,984 (57.60)	2,791 (55.68)	845 (52.39)	330 (56.90)	284 (52.59)	313 (62.73)	103 (52.82)
**Tumor size, cm**								
<3	7,861 (26.84)	6,497 (26.76)	1,364 (27.21)	432 (26.78)	171 (29.48)	172 (31.85)	105 (21.04)	56 (28.72)
3–5	12,557 (42.87)	10,681 (44.00)	1,876 (37.42)	638 (39.55)	221 (38.10)	191 (35.37)	177 (35.47)	78 (40.00)
>5	8,872 (30.29)	7,099 (29.24)	1,773 (35.37)	543 (33.66)	188 (32.41)	177 (32.78)	217 (43.49)	61 (31.28)
**Lymph nodes examined**								
<12	10,891 (37.18)	8,705 (35.86)	2,186 (43.61)	703 (43.58)	285 (49.14)	251 (46.48)	214 (42.89)	80 (41.03)
≥12	18,399 (62.82)	15,572 (64.14)	2,827 (56.39)	910 (56.42)	295 (50.86)	289 (53.52)	285 (57.11)	115 (58.97)
**Histology**				
Adenocarcinoma	26,470 (90.37)	21,977 (90.53)	4,493 (89.63)	1,448 (89.77)	525 (90.52)	490 (90.74)	432 (86.57)	178 (91.28)
Other	2,820 (9.63)	2,300 (9.47)	520 (10.37)	165 (10.23)	55 (9.48)	50 (9.26)	67 (13.43)	17 (8.72)
**Tumor grade**								
I–II	24,975 (85.27)	20,702 (85.27)	4,273 (85.24)	1,380 (85.55)	498 (85.86)	452 (83.70)	433 (86.77)	162 (83.08)
III–IV	4,315 (14.73)	3,575 (14.73)	740 (14.76)	233 (14.45)	82 (14.14)	88 (16.30)	66 (13.23)	33 (16.92)
**Stage**								
I	7,545 (25.76)	6,187 (25.49)	1,358 (27.09)	436 (27.03)	186 (32.07)	149 (27.59)	115 (23.05)	48 (24.62)
II	12,140 (41.45)	9,947 (40.97)	2,193 (43.75)	685 (42.47)	253 (43.62)	243 (45.00)	246 (49.30)	93 (47.69)
III	9,605 (32.79)	8,143 (33.54)	1,462 (29.16)	492 (30.50)	141 (24.31)	148 (27.41)	138 (27.66)	54 (27.69)
**Chemotherapy**								
No/Unknown	19,033 (64.98)	15,633 (64.39)	3,400 (67.82)	1,072 (66.46)	414 (71.38)	374 (69.26)	341 (68.34)	122 (62.56)
Yes	10,257 (35.02)	8,644 (35.61)	1,613 (32.18)	541 (33.54)	166 (28.62)	166 (30.74)	158 (31.66)	73 (37.44)
**Surgery**								
Partial colectomy	14,431 (49.27)	12,003 (49.44)	2,428 (48.43)	754 (46.75)	291 (50.17)	263 (48.70)	246 (49.30)	91 (46.67)
Subtotal or total colectomy	14,859 (50.73)	12,274 (50.56)	2,585 (51.57)	859 (53.25)	289 (49.83)	277 (51.30)	253 (50.70)	104 (53.33)
**Length of follow-up, years**								
5–10	16,313 (55.69)	13,823 (56.94)	2,490 (49.67)	596 (36.95)	371 (63.97)	223 (41.30)	268 (53.71)	73 (37.44)
10–15	9,655 (32.96)	7,831 (32.26)	1,824 (36.39)	683 (42.34)	176 (30.34)	233 (43.15)	165 (33.07)	87 (44.62)
15–19	3,322 (11.34)	2,623 (10.80)	699 (13.94)	334 (20.71)	33 (5.69)	84 (15.56)	66 (13.23)	35 (17.95)
**SPM time, years**	/	/	5.3 (2.8–8.3)	4.8 (2.3–7.5)	6.0 (4.1–9.0)	5.5 (3.1–8.4)	4.0 (1.8–7.3)	6.0 (2.8–8.9)
**Status**								
Alive	21,029 (71.80)	18,321 (75.47)	2,708 (54.02)	1,133 (70.24)	168 (28.97)	322 (59.63)	274 (54.91)	134 (68.72)
Dead	8,261 (28.20)	5,956 (24.53)	2,305 (45.98)	480 (29.76)	412 (71.03)	218 (40.37)	225 (45.09)	61 (31.28)
**Cause of death**								
First primary cancer	2,766 (33.48)	2,169 (36.42)	597 (25.90)	97 (20.21)	52 (12.62)	28 (12.84)	139 (61.78)	9 (14.75)
Multiple malignancies	1,019 (12.34)	/	1,019 (44.21)	167 (34.79)	292 (70.87)	80 (36.70)	17 (7.56)	20 (32.79)
Noncancer cause	4,476 (54.18)	3,787 (63.58)	689 (29.89)	216 (45.00)	68 (16.50)	110 (50.46)	69 (30.67)	32 (52.46)

*OOPM, only one primary malignancy; SPM, secondary primary malignancy*.

**Table 2:** Clinicopathological variables of female CC survivors with stratified events.

**Risk factors**				**Top 5 SPM in female**, ***n*** **(%)**				
	**Overall, *n* (%)**	**OOPM cohort, *n* (%)**	**SPM cohort, *n* (%)**	**Breast**	**Lung and Bronchus**	**Colon**	**Uterus**	**Urinary Bladder and Kidney**
	**(27,640)**	**(24,048)**	**(3,592)**	**(926)**	**(489)**	**(416)**	**(296)**	**(201)**
**Age at initial diagnosis, years**								
18–49	3,707 (13.41)	3,396 (14.12)	311 (8.66)	75 (8.10)	12 (2.45)	30 (7.21)	66 (22.30)	13 (6.47)
50–64	9,974 (36.09)	8,825 (36.70)	1,149 (31.99)	308 (33.26)	160 (32.72)	103 (24.76)	121 (40.88)	70 (34.83)
65–79	13,959 (50.50)	11,827 (49.18)	2,132 (59.35)	543 (58.64)	317 (64.83)	283 (68.03)	109 (36.82)	118 (58.71)
**Race**								
White	21,368 (77.31)	18,500 (76.93)	2,868 (79.84)	753 (81.32)	406 (83.03)	329 (79.09)	222 (75.00)	159 (79.10)
Black	3,483 (12.60)	3,049 (12.68)	434 (12.08)	106 (11.45)	53 (10.84)	52 (12.50)	39 (13.18)	30 (14.93)
Other	2,789 (10.09)	2,499 (10.39)	290 (8.07)	67 (7.24)	30 (6.13)	35 (8.41)	35 (11.82)	12 (5.97)
**Marital status**
Married	15,746 (56.97)	13,793 (57.36)	1,953 (54.37)	531 (57.34)	249 (50.92)	223 (53.61)	160 (54.05)	118 (58.71)
Unmarried	11,894 (43.03)	10,255 (42.64)	1,639 (45.63)	395 (42.66)	240 (49.08)	193 (46.39)	136 (45.95)	83 (41.29)
**Initial diagnosed site**								
Right colon	13,134 (47.52)	11,300 (46.99)	1,834 (51.06)	484 (52.27)	265 (54.19)	215 (51.68)	147 (49.66)	110 (54.73)
Left colon	14,506 (52.48)	12,748 (53.01)	1,758 (48.94)	442 (47.73)	224 (45.81)	201 (48.32)	149 (50.33)	91 (45.27)
**Tumor size, cm**								
<3	7,599 (27.49)	6,689 (27.82)	910 (25.33)	239 (25.81)	119 (24.34)	90 (21.63)	54 (18.24)	58 (28.86)
3–5	12,679 (45.87)	11,010 (45.78)	1,669 (46.46)	458 (49.46)	212 (43.35)	174 (41.83)	127 (42.91)	101 (50.25)
>5	7,362 (26.64)	6,349 (26.40)	1,013 (28.20)	229 (24.73)	158 (32.31)	152 (36.54)	115 (38.85)	42 (20.90)
**Lymph nodes examined**								
<12	9,569 (34.62)	8,177 (34.00)	1,392 (38.75)	363 (39.20)	215 (43.97)	151 (36.30)	95 (32.09)	88 (43.78)
≥12	18,071 (65.38)	15,871 (66.00)	2,200 (61.25)	563 (60.80)	274 (56.03)	265 (63.70)	201 (67.91)	113 (56.22)
**Histology**								
Adenocarcinoma	24,715 (89.42)	21,562 (89.66)	3,153 (87.78)	812 (87.69)	411 (84.05)	359 (86.30)	258 (87.16)	183 (91.04)
Other	2,925 (10.58)	2,486 (10.34)	439 (12.22)	114 (12.31)	78 (15.95)	57 (13.70)	38 (12.84)	18 (8.96)
**Tumor grade**								
I–II	23,093 (83.55)	20,169 (83.87)	2,924 (81.40)	763 (82.40)	394 (80.57)	332 (79.81)	239 (80.74)	161 (80.10)
III–IV	4,547 (16.45)	3,879 (16.13)	668 (18.60)	163 (17.60)	95 (19.43)	84 (20.19)	57 (19.26)	40 (19.90)
**Stage**								
I	6,880 (24.89)	6,002 (24.96)	878 (24.44)	236 (25.49)	109 (22.29)	86 (20.67)	58 (19.59)	53 (26.37)
II	11,453 (41.44)	9,835 (40.90)	1,618 (45.04)	412 (44.49)	240 (49.08)	205 (49.28)	144 (48.65)	89 (44.28)
III	9,307 (33.67)	8,211 (34.14)	1,096 (30.51)	278 (30.02)	140 (28.63)	125 (30.05)	94 (31.76)	59 (29.35)
**Chemotherapy**								
No/Unknown	17,681 (63.97)	15,372 (63.92)	2,309 (64.28)	626 (67.60)	305 (62.37)	271 (65.14)	179 (60.47)	125 (62.19)
Yes	9,959 (36.03)	8,676 (36.08)	1,283 (35.72)	300 (32.40)	184 (37.63)	145 (34.86)	117 (39.53)	76 (37.81)
**Surgery**								
Partial colectomy	13,136 (47.53)	11,479 (47.73)	1,657 (46.13)	425 (45.90)	195 (39.88)	191 (45.91)	135 (45.61)	102 (50.75)
Subtotal or total colectomy	14,504 (52.47)	12,569 (52.27)	1,935 (53.87)	501 (54.10)	294 (60.12)	225 (54.09)	161 (54.39)	99 (49.25)
**Length of follow-up, years**								
5–10	14,521 (52.54)	12,807 (53.26)	1,714 (47.72)	345 (37.26)	283 (57.87)	224 (53.85)	119 (40.20)	98 (48.76)
10–15	9,773 (35.36)	8,406 (34.96)	1,367 (38.06)	413 (44.60)	162 (33.13)	136 (32.69)	118 (39.86)	67 (33.33)
15–19	3,346 (12.11)	2,835 (11.79)	511 (14.23)	168 (18.14)	44 (9.00)	56 (13.46)	59 (19.93)	36 (17.91)
**SPM time, years**	/	/	5.5 (2.8–8.5)	5.3 (2.8–8.3)	6.4 (4.4–9.0)	4.2 (2.0–7.3)	5.0 (2.3–8.4)	4.8 (2.0–7.8)
**Status**								
Alive	20,920 (75.69)	18,852 (78.39)	2,068 (57.57)	681 (73.54)	167 (34.15)	242 (58.17)	210 (70.95)	124 (61.69)
Dead	6,720 (24.31)	5,196 (21.61)	1,524 (42.43)	245 (26.46)	322 (65.85)	174 (41.83)	86 (29.05)	77 (38.31)
**Cause of death**								
First primary cancer	2,095 (31.18)	1,726 (33.22)	369 (24.21)	37 (15.10)	52 (16.15)	72 (41.38)	14 (16.28)	9 (11.69)
Multiple malignancies	701 (10.43)	/	701 (46.00)	93 (37.96)	221 (68.63)	18 (10.34)	42 (48.84)	38 (49.35)
Noncancer cause	3,924 (58.39)	3,470 (66.78)	454 (29.79)	115 (46.94)	49 (15.22)	84 (48.28)	30 (34.88)	30 (38.96)

*OOPM, only one primary malignancy; SPM, secondary primary malignancy*.

### Survival Analysis and Cumulative Incidence of a SPM

**Figure 1:**
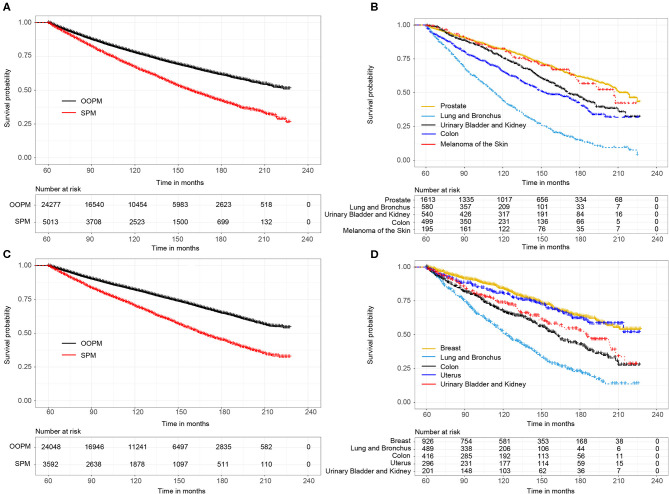
**(A)** Overall survival (OS) between male survivors with and without second primary malignancies (SPMs) based on the Kaplan-Meier method; **(B)** OS between male survivors with top 5 SPMs sites; **(C)** OS between female survivors with and without SPMs; **(D)** OS between female survivors with top 5 SPMs sites.

For male survivors, the median OS of the SPM cohort was 13.2 years, while the OOPM cohort did not reached the median survival time (Figure 1A). Subgroup analysis showed that the median OS of survivors with top 5 SPM was 18.2 years (prostate), 9.5 years (lung and bronchus), 14.3 years (urinary bladder and kidney), 11.8 years (colon), and 17.3 years (melanoma of the skin), respectively (Figure 1B). Similarly, the SPM cohort, of which the median OS was 13.8 years, showed worse prognosis than the OOPM cohort (Figure 1C). The median OS of female survivors with top 5 SPM was not reached (breast), 10.3 years (lung and bronchus), 13.5 years (colon), not reached (uterus), and 15.4 years (urinary bladder and kidney), respectively (Figure 1D).

**Figure 2:**
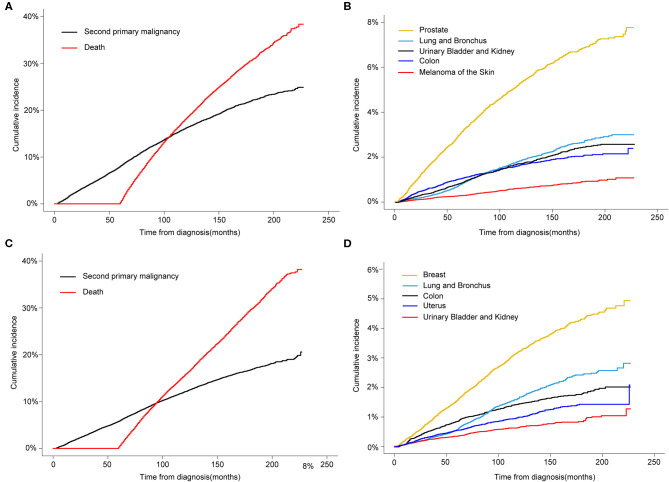
**(A)** Cumulative incidence of second primary malignancy (SPM) and death in male survivors based on the Gray method; **(B)** Cumulative incidence of top 5 SPMs sites in male survivors; **(C)** Cumulative incidence of SPM and death in female survivors; **(D)** Cumulative incidence of top 5 SPMs sites in female survivors.

Regarding the death as a competing factor, the 3-, 5-, and 10-year cumulative incidence of a SPM in male survivors were 4.69, 8.07, and 16.23%, respectively (Figure 2A). Especially, 1.80, 2.94, and 5.32% male survivors occurred a second primary prostate carcinoma in the 3-, 5-, and 10-year, respectively (Figure 2B). The 3-, 5-, and 10-year cumulative incidence of a SPM in female survivors were 3.46, 5.81, and 12.11%, respectively (Figure 2C). Of these patients, 0.89, 1.55, and 3.23% survivors suffered from a second primary breast carcinoma in the 3-, 5-, and 10-year, respectively (Figure 2D).

### Unbiased Risk Factors of Developing a SPM

Risk predictors for developing a SPM after the initial CC diagnosis were estimated by using the Fine and Gray method, and the results of the characteristics were provided in Tables 3, 4. Older age, black race, married status, tumor size > 5 cm and number of lymph nodes examined <12 were significantly related to a higher risk of developing a SPM or a second primary prostate carcinoma in male survivors. For female survivors, older age, right colon, tumor size > 5 cm, tumor grade III–IV, stage I–II and chemotherapy were significantly linked to an increased risk of developing a SPM, while only three variables including age at initial diagnosis, race and initial diagnosed site showed significant difference in developing a second primary breast carcinoma, which might be not suitable for conducting the nomogram.

**Table 3:** Risk factors associated with development of second primary malignancy by organ sites in male survivors.

**Variable**	**Overall**		**Prostate**		**Lung and Bronchus**	**Urinary Bladder and Kidney**	**Colon**	**Melanoma of the Skin**
	**SHR (95%CI)**	***P*-value**	**SHR (95%CI)**	***P*-value**	**SHR (95%CI)**	**SHR (95%CI)**	**SHR (95%CI)**	**SHR (95%CI)**
**Age at initial diagnosis, years**								
18–49	Ref		Ref		Ref	Ref	Ref	Ref
50–64	1.98 (1.75–2.23)	<0.001	3.87 (2.93–5.13)	<0.001	3.00 (1.87–4.82)	3.26 (2.00–5.29)	/	1.88 (1.04–3.39)
65–79	2.85 (2.53–3.21)	<0.001	5.33 (4.03–7.03)	<0.001	5.74 (3.61–9.11)	5.06 (3.13–8.17)	1.41 (1.06–1.88)	2.30 (1.28–4.14)
**Race**								
White	Ref		Ref		Ref	Ref	Ref	Ref
Black	1.21 (1.1–1.33)	<0.001	1.98 (1.74–2.26)	<0.001	/	0.66 (0.47–0.93)	/	0.08 (0.02–0.33)
Other	0.80 (0.72–0.89)	<0.001	0.64 (0.52–0.80)	<0.001	/	0.68 (0.48–0.96)	/	0.05 (0.01–0.35)
**Marital status**								
Married	Ref		Ref		Ref	Ref	Ref	Ref
Unmarried	0.93 (0.87–1.00)	0.047	0.80 (0.71–0.90)	<0.001	/	/	/	/
**Initial diagnosed site**								
Right colon	Ref		Ref		Ref	Ref	Ref	Ref
Left colon	0.97 (0.91–1.04)	0.440	0.84 (0.74–0.94)	0.003	/	0.82 (0.67–0.99)	1.44 (1.16–1.80)	/
**Tumor size, cm**								
<3	Ref		Ref		Ref	Ref	Ref	Ref
3–5	0.89 (0.82–0.96)	0.002	0.99 (0.87–1.13)	0.870	/	0.72 (0.58–0.90)	/	/
>5	1.35 (1.25–1.47)	<0.001	1.33 (1.14–1.54)	<0.001	1.35 (1.06–1.72)	/	2.02 (1.53–2.68)	/
**Lymph nodes examined**								
<12	Ref		Ref		Ref	Ref	Ref	Ref
≥12	0.89 (0.84–0.95)	<0.001	0.83 (0.75–0.93)	0.001	0.74 (0.62–0.88)	0.78 (0.65–0.93)	0.82 (0.68–0.99)	/
**Histology**								
Adenocarcinoma	Ref		Ref		Ref	Ref	Ref	Ref
Other	1.01 (0.92–1.11)	0.830	1.02 (0.86–1.20)	0.830	/	/	1.35 (1.03–1.77)	/
**Tumor grade**								
I–II	Ref		Ref		Ref	Ref	Ref	Ref
III–IV	1.02 (0.94–1.10)	0.650	1.00 (0.87–1.16)	0.970	/	/	/	/
**Stage**								
I	Ref		Ref		Ref	Ref	Ref	Ref
II	0.93 (0.86–1.01)	0.078	0.92 (0.80–1.06)	0.260	/	/	/	/
III	0.84 (0.77–0.92)	<0.001	0.89 (0.75–1.05)	0.160	0.62 (0.47–0.83)	/	/	/
**Chemotherapy**								
No/Unknown	Ref		Ref		Ref	Ref	Ref	Ref
Yes	1.03 (0.96–1.11)	0.440	1.10 (0.96–1.24)	0.160	/	/	/	/
**Surgery**								
Partial colectomy	Ref		Ref		Ref	Ref	Ref	Ref
Subtotal or total colectomy	0.99 (0.93–1.06)	0.870	0.97 (0.86–1.09)	0.580	/	/	/	/

*SHR, Subdistribution hazard ratio; 95%CI, 95% confidence interval. For the second primary malignancy sites of lung and bronchus, urinary bladder and kidney, colon and melanoma of the skin, significant SHR and 95%CI was exhibited in the table*.

**Table 4:** Risk factors associated with development of second primary malignancy by organ sites in female survivors.

**Variable**	**Overall**		**Breast**		**Lung and Bronchus**	**Colon**	**Uterus**	**Urinary Bladder and Kidney**
	**SHR (95%CI)**	***P*-value**	**SHR (95%CI)**	***P*-value**	**SHR (95%CI)**	**SHR (95%CI)**	**SHR (95%CI)**	**SHR (95%CI)**
**Age at initial diagnosis, years**								
18–49	Ref		Ref		Ref	Ref	Ref	Ref
50–64	1.40 (1.24–1.59)	<0.001	1.49 (1.16–1.93)	0.002	5.05 (2.81–9.08)	/	0.71 (0.52–0.97)	1.94 (1.07–3.52)
65–79	1.80 (1.59–2.04)	<0.001	1.85 (1.45–2.38)	<0.001	6.92 (3.88–12.34)	2.68 (1.83–3.92)	0.44 (0.31–0.62)	2.39 (1.33–4.30)
**Race**								
White	Ref		Ref		Ref	Ref	Ref	Ref
Black	0.98 (0.88–1.08)	0.630	0.88 (0.71–1.08)	0.210	/	/	/	/
Other	0.82 (0.73–0.93)	0.001	0.74 (0.58–0.95)	0.020	0.66 (0.46–0.96)	/	/	/
**Marital status**								
Married	Ref		Ref		Ref	Ref	Ref	Ref
Unmarried	1.07 (1.00–1.14)	0.059	0.96 (0.84–1.09)	0.520	1.22 (1.02–1.46)	/	/	/
**Initial diagnosed site**								
Right colon	Ref		Ref		Ref	Ref	Ref	Ref
Left colon	0.92 (0.85–1.00)	0.048	0.86 (0.73–1.00)	0.049	/	/	/	0.64 (0.45–0.89)
**Tumor size, cm**								
<3	Ref		Ref		Ref	Ref	Ref	Ref
3–5	1.09 (1.00–1.19)	0.040	1.18 (0.99–1.40)	0.057	/	/	/	/
>5	1.13 (1.02–1.24)	0.021	1.00 (0.81–1.23)	1.000	1.31 (1.00–1.7)	1.65 (1.24–2.18)	1.91 (1.31–2.77)	/
**Lymph nodes examined**								
<12	Ref		Ref		Ref	Ref	Ref	Ref
≥12	0.99 (0.92–1.06)	0.710	0.92 (0.80–1.05)	0.230	0.73 (0.60–0.88)	/	/	/
**Histology**								
Adenocarcinoma	Ref		Ref		Ref	Ref	Ref	Ref
Other	1.08 (0.97–1.19)	0.150	1.11 (0.91–1.36)	0.300	1.37 (1.07–1.75)	/	/	/
**Tumor grade**								
I–II	Ref		Ref		Ref	Ref	Ref	Ref
III–IV	1.14 (1.04–1.24)	0.003	1.07 (0.90–1.28)	0.420	/	/	/	/
**Stage**								
I	Ref		Ref		Ref	Ref	Ref	Ref
II	0.99 (0.90–1.08)	0.800	1.03 (0.86–1.24)	0.720	/	/	/	/
III	0.83 (0.74–0.92)	0.001	0.95 (0.77–1.18)	0.650	/	/	/	/
**Chemotherapy**								
No/Unknown	Ref		Ref		Ref	Ref	Ref	Ref
Yes	1.16 (1.07–1.26)	<0.001	0.94 (0.79–1.11)	0.440	1.39 (1.11–1.75)	/	/	1.42 (1.01–2.00)
**Surgery**								
Partial colectomy	Ref		Ref		Ref	Ref	Ref	Ref
Subtotal or total colectomy	0.96 (0.89–1.04)	0.280	0.95 (0.81–1.11)	0.510	1.24 (1.01–1.53)	/	/	0.70 (0.51–0.98)

*SHR, Subdistribution hazard ratio; 95%CI, 95% confidence interval. For the second primary malignancy sites of lung and bronchus, colon, uterus, urinary bladder and kidney, significant SHR and 95%CI was exhibited in the table*.

### Competing-Risk Nomogram Construction and Validation

**Figure 3:**
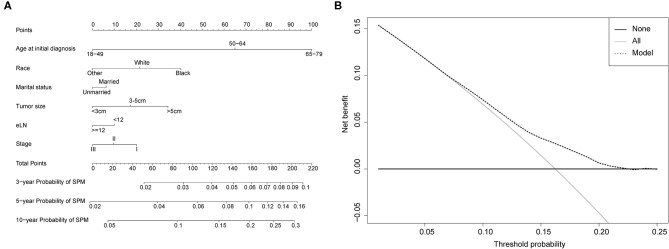
**(A)** Competing-risk nomogram for predicting the 3-, 5-, and 10-year risk of developing second primary malignancy (SPM) in male survivors. There are six factors in this nomogram, including age at initial diagnosis, race, marital status, number of lymph nodes examined (eLN) and stage; **(B)** AS shown, if the threshold probability was between 1 and 22%, then using the nomogram to predict the probability of developing SPM added more clinical benefits.

**Figure 4:**
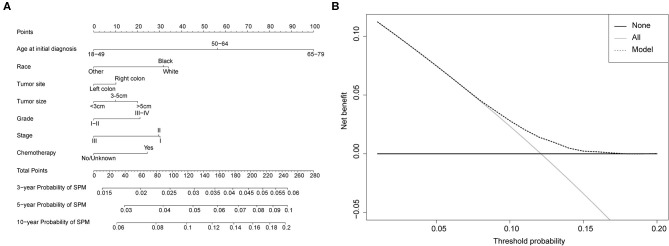
**(A)** Competing-risk nomogram for predicting the 3-, 5-, and 10-year risk of developing second primary malignancy (SPM) in female survivors. There are seven factors in this nomogram, including age at initial diagnosis, race, tumor site, tumor size, grade, stage and chemotherapy; **(B)** AS shown, if the threshold probability was between 1 and 17%, then using the nomogram to predict the probability of developing SPM added more clinical benefits.

**Figure 5:**
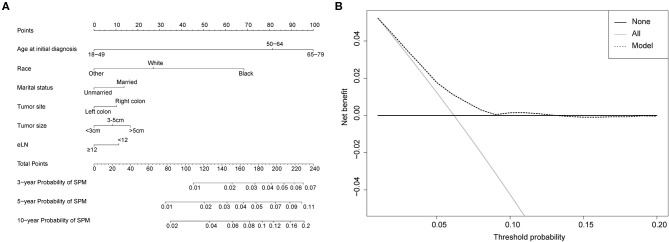
**(A)** Competing-risk nomogram for predicting the 3-, 5-, and 10-year risk of developing a second prostate carcinoma in male survivors. There are six factors in this nomogram, including age at initial diagnosis, race, marital status, tumor site, tumor size and number of lymph nodes examined (eLN); **(B)** AS shown, if the threshold probability was between 1 and 13%, then using the nomogram to predict the probability of developing SPM added more clinical benefits.

Competing-risk nomograms were established based on the previously mentioned factors to predict for developing a SPM in male survivors (Figure 3A) or female survivors (Figure 4A), and for developing a second prostate carcinoma in male survivors (Figure 5A). The risk scores of these variables were calculated in Supplementary Table 1. Low-, medium-, and high-risk survivors were identified using the 25th and 75th percentile values of the risk score. Compared with the low-risk group, the high-risk group exhibited a significantly higher cumulative incidence in 3-, 5-, and 10-year after the initial diagnosis (Supplementary Figures 2–4). The C-index of three competing-risk nomograms was 59.8, 56.8, and 63.7%, respectively, and the calibration curves revealed relatively excellent agreement between the nomogram prediction and the actual observation (Supplementary Figures 5–7). Furthermore, DCAs were performed on the competing-risk nomograms, indicating that there were proper threshold probabilities for predicting a SPM in male or female survivors (Figures 3B, 4B), and for predicting a second primary prostate in male survivors (Figure 5B). Although no nomogram was established before for predicting SPM probability in colon cancer survivors underwent colectomy, we compared the nomograms with the model involving some variables stratified by demographic characteristics (age, race, marital status) and tumor covariates (tumor size, tumor site, grade, stage, etc.). The results showed that the C-index of demographic characteristics (0.587 of male survivors, 0.558 of female survivors) and tumor covariates (0.536 of male survivors, 0.532 of female survivors) in competing-risk nomograms were all smaller than the nomograms both involving demographic characteristics and tumor covariates (0.598 of male survivors, 0.568 of female survivors). Similarly, our model for predicting a second primary prostate carcinoma (C-index = 0.637) was more reliable than that of demographic characteristics (C-index = 0.628) and tumor covariates (C-index = 0.546). These results revealed that demographic characteristics played a key role in identifying SPM.

## Discussion

In fact, the number of cancer survivors in the U.S. is estimated to elevate to about 20 million by 2024 (5), and 17–19% of all new primary malignancies occurred in these survivors (17) of cancer. However, few studies focus on colon cancer survivors with SPMs, and most of these studies resulted in inaccurate estimates using logistic regression (10, 18) or Cox regression model (19), in which the death is not considered as a competing event. Recently, Jia et al. studied the risk of second primary malignancies in patients with colorectal cancer and found that male or colon cancer survivors were prone to occurring SPMs, but the risk of the specific sites of SPMs was not involved (20). Thus, the current study aimed to identify the risk factors of the top 5 SPMs in male and female survivors with colon cancer, respectively. We ensured a long-term follow-up time after the initial diagnosis by screening patients from the latest population-based database between 1998 and 2011, subsequently, and to identify CC survivors with a SPM, regarding the death as a competing factor. Furthermore, nomograms were established to intuitively display the 3-, 5-, and 10-year prediction of SPM probabilities in male and female CC patients in this study, respectively.

Some interesting findings were confirmed in the present study. Firstly, 17.1% of male survivors and 13.0% female survivors suffered from a SPM with a follow-up time of at least 5 years. Hence, it is significant to pay more attention to profile the characteristics of cancer survivors, as these traits might determine their health in future. Additionally, prostate and breast were the most common SPMs sites in male and female survivors, respectively, which is consistent with the estimated new cancer cases in 2020 (3). Ten-year cumulative incidence of the former was 5.32% in male while that of the latter was 3.23% in female. Further details of clinical and survival information about CC patients with SPMs were exhibited in this study. We also found that CC patients with only one primary malignancy experienced better prognosis than those with SPMs, regardless of gender. In addition, subgroup analysis of these with top 5 SPMs showed that lung and bronchus was the most fatal SPM site for both male and female survivors, with the consistence of most leading cancer type for deaths (3). Male patients with a second primary prostate carcinoma and female patients with a second primary breast carcinoma showed the best survival among top 5 SPMs sites. Therefore, it is necessary for CC survivors at high risk of SPMs to receive intensive and long-term surveillance, especially for a second primary prostate carcinoma in male patients and breast cancer in female survivors.

Secondly, identifying the comprehensive effects of demographic and tumor factors related to the risk of developing SPMs, attracts growing concern for CC survivor surveillance and management. As showed in our study, cumulative incidence of the death excessed that of SPM ~8 years after the initial diagnosis in both male and female survivors, indicating that a large proportion of CC patients die before the development of a SPM and it is necessary to consider the death as a competing event. Hence, multivariable competing risk models were conducted to analyze the risk of SPMs in male or female survivors. Our multivariable competing risk models showed that male CC survivors with SPMs were prone to being older, black in race, married status, tumor size > 5 cm, number of lymph nodes examined <12, and stage I-II, while older age, right colon, bigger tumor size, tumor grade III-IV, stage I-II, and chemotherapy were significantly linked to a higher risk of developing a SPM for female survivors. Especially, the risk of a second primary prostate carcinoma in black male patients was higher than that in white race [subdistribution hazard ratio (SHR) = 1.98, 95%CI = 1.74–2.26], and male survivors aged 65–79 years has more than 5 times higher risk than those aged 18–49 years. By comparing the C-index among the models, we found that demographic factors (age, race and marital status) were crucial to identify the risk of developing a SPM. Older age may lead to immunosenescence in these survivors (21), increasing the second cancer disease (22, 23). Interestingly, like the results of previous studies in other tumors (24, 25), black race increases the risk of SPMs in male CC survivors, especially for a second primary prostate carcinoma (3). We unexpectedly found higher risk to develop a SPM for married male CC patients, probably resulting from the impact of marital status on the occurrence of prostate carcinoma, and similar results showed that older, separated or divorced men existed a decreased risk for the development of prostate cancer (26), which is not easily explained. A meta-analysis might reveal the potential role of marital status to the cancer risk, for that men with fewer sexual partner numbers were associated with a significantly decreased risk of prostate cancer (27) and married men may have more sexual activities.

Additionally, tumor covariates (tumor size, tumor site, grade, stage, etc.) also were independent risk factor to determine the status of SPM, although their effects were relatively limited. It was reported that the location of colon cancer contributed to different incidence of second primary gastrointestinal malignancies, and the standardized incidence ratio of small intestinal cancer was higher in right colon cancer than that in left colon cancer (28). However, in our study, only female survivors with right colon cancer showed increased risk when compared to those with left colon cancer, and *P*-value is 0.048, showing the unstable statistical effect. The relationship between SPM risk and other tumor covariates (tumor size, stage, grade and number of lymph nodes examined) was not reported before. Our results revealed that the size of initial tumor was positively correlated with the incidence of SPMs, especially for developing a second colon cancer, which might be due to non-standard resection. Survivors with stage I-II usually experienced better prognosis and had enough follow-up time to develop more SPMs, so their risk was increasing. Nevertheless, there were some phenomena that are difficult to explain and these need to be further researched: (1) Female patients with grade III-IV were at higher risk; (2) Number of lymph nodes examined <12 in male survivors was an independent risk factor; (3) Chemotherapy could increase the SPM risk for female patients, mainly including a SPM site of lung and bronchus or urinary bladder and kidney.

Finally, regarding the sample size and the practicality of the models, we developed three competing-risk nomograms for predicting SPM risk of male or female survivors, and for predicting the probability of occurring a second primary prostate in male survivors. These nomograms could be effective for using common characteristics of CC patients to predict the 3-, 5-, and 10-year incidence probabilities of the outcome after the initial cancer diagnosis, considering the death as a competing factor. Moreover, the evaluation of our nomograms was demonstrated to have relatively high discrimination and calibration, and to reveal good clinical utility in the proper threshold probability range, which could help doctors identify CC survivors at the high risk to develop new primary cancers.

There are still some limitations in our study. First, this was a population-based retrospective analysis using the SEER database lacking some important risk factors for SPMs, such diet habit and lifestyle, family history of cancer, chemoradiotherapy protocols, and oncogene testing. It would be better to include the above variables in these three models, which might be the future direction of prediction for SPMs. Second, it is hard to distinguish the initial simultaneous cancers within a standard of 2-month interval or to identify metastases from SPMs, leading to the wrong estimates for the risk probabilities of developing SPMs. Of course, the diagnosis time of a SPM is also not the accurate time when it occurred, and with the development of rigorous surveillance and detection technology, these problems would be solved effectively. Finally, our models still need to be verified by external populations, although its internal validation showed good consistency. Risk factors for various SPMs sites of CC survivors need to be further studied to improve better surveillance strategies.

## Conclusion

We profiled the characteristics of colon cancer survivors treated with colectomy and found that prostate cancer in male and breast cancer in female were the most common SPMs sites, respectively. Older age, black race in men and married male are the independent risk factors for developing SPMs. Subsequently, we established nomograms to predict the SPM probabilities and identify high-risk population. Therefore, it is suggested for these high-risk survivors that during postoperative follow-up of colon cancer, men can appropriately participate in prostate-specific antigen (PSA) examination, while women can appropriately participate in mammography examination.

## Data Availability Statement

Publicly available datasets were analyzed in this study. This data can be found here: https://seer.cancer.gov/.

## Ethics Statement

No ethical approval was sought for this study, as the data used were collected from the public SEER database, which is available as open-access and anonymized data.

## Author Contributions

BZ, KG, and SR conceived and designed the study, performed the study, analyzed the data, prepared figures and/or tables, and authored or reviewed drafts of the paper. XZ and MS conceived and designed the study, performed the study, analyzed the data. BZ, KG, and LS performed the study and authored or reviewed drafts of the paper. SR conceived and designed the study, performed the study, authored or reviewed drafts of the paper, and approved the final draft. All authors contributed to the article and approved the submitted version.

## Conflict of Interest

The authors declare that the research was conducted in the absence of any commercial or financial relationships that could be construed as a potential conflict of interest.

## Supplementary Material

Supplementary Figure 1 The flowchart of cases selection.

Supplementary Figure 2 The 3-, 5-, and 10-year cumulative incidence rates of SPMs among low-, medium-, and high-risk groups of male survivors. Risk stratification was conducted by the 25th and 75th percentile values of the risk score in the nomogram, which is 113 and 155, respectively. The cumulated incidence rates of these groups were assessed using the Fine-Gray method. Compared with the low-risk group (light-blue bar), the high-risk group (orange bar) exhibited a significantly higher cumulated incidence in 3-, 5-, and 10-year after the initial diagnosis.

Supplementary Figure 3 The 3-, 5-, and 10-year cumulative incidence rates of SPMs among low-, medium-, and high-risk groups of female survivors. Risk stratification was conducted by the 25th and 75th percentile values of the risk score in the nomogram, which is 124 and 178, respectively. The cumulated incidence rates of these groups were assessed using the Fine-Gray method. Compared with the low-risk group (light-blue bar), the high-risk group (orange bar) exhibited a significantly higher cumulated incidence in 3-, 5-, and 10-year after the initial diagnosis.

Supplementary Figure 4 The 3-, 5-, and 10-year cumulative incidence rates of second prostate carcinoma among low-, medium-, and high-risk groups of male survivors. Risk stratification was conducted by the 25th and 75th percentile values of the risk score in the nomogram, which is 126 and 159, respectively. The cumulated incidence rates of these groups were assessed using the Fine-Gray method. Compared with the low-risk group (light-blue bar), the high-risk group (orange bar) exhibited a significantly higher cumulated incidence in 3-, 5-, and 10-year after the initial diagnosis.

Supplementary Figure 5 Calibration curves for 3-year prediction (black line), 5-year prediction (blue line) and 10-year prediction (orange line) in male survivors. X-axis: predicted probability of developing second primary malignancy based on the multivariate competing risk model. Y-axis: Observed cumulative probability.

Supplementary Figure 6 Calibration curves for 3-year prediction (black line), 5-year prediction (blue line), and 10-year prediction (orange line) in female survivors. X-axis: predicted probability of developing second primary malignancy based on the multivariate competing risk model. Y-axis: Observed cumulative probability.

Supplementary Figure 7 Calibration curves for 3-year prediction (black line), 5-year prediction (blue line), and 10-year prediction (orange line) in male survivors. X-axis: predicted probability of developing second prostate carcinoma based on the multivariate competing risk model. Y-axis: Observed cumulative probability.

Supplementary Table 1 Point assignment and risk score in the nomograms.
