# Supplementary figures and images for: Risk of Second Primary Malignancies in Colon Cancer Patients Treated With Colectomy
Source: Front Oncol. 2020 Jul 16;10:1154. doi: 10.3389/fonc.2020.01154 (PMC7378742; doi:10.3389/fonc.2020.01154)

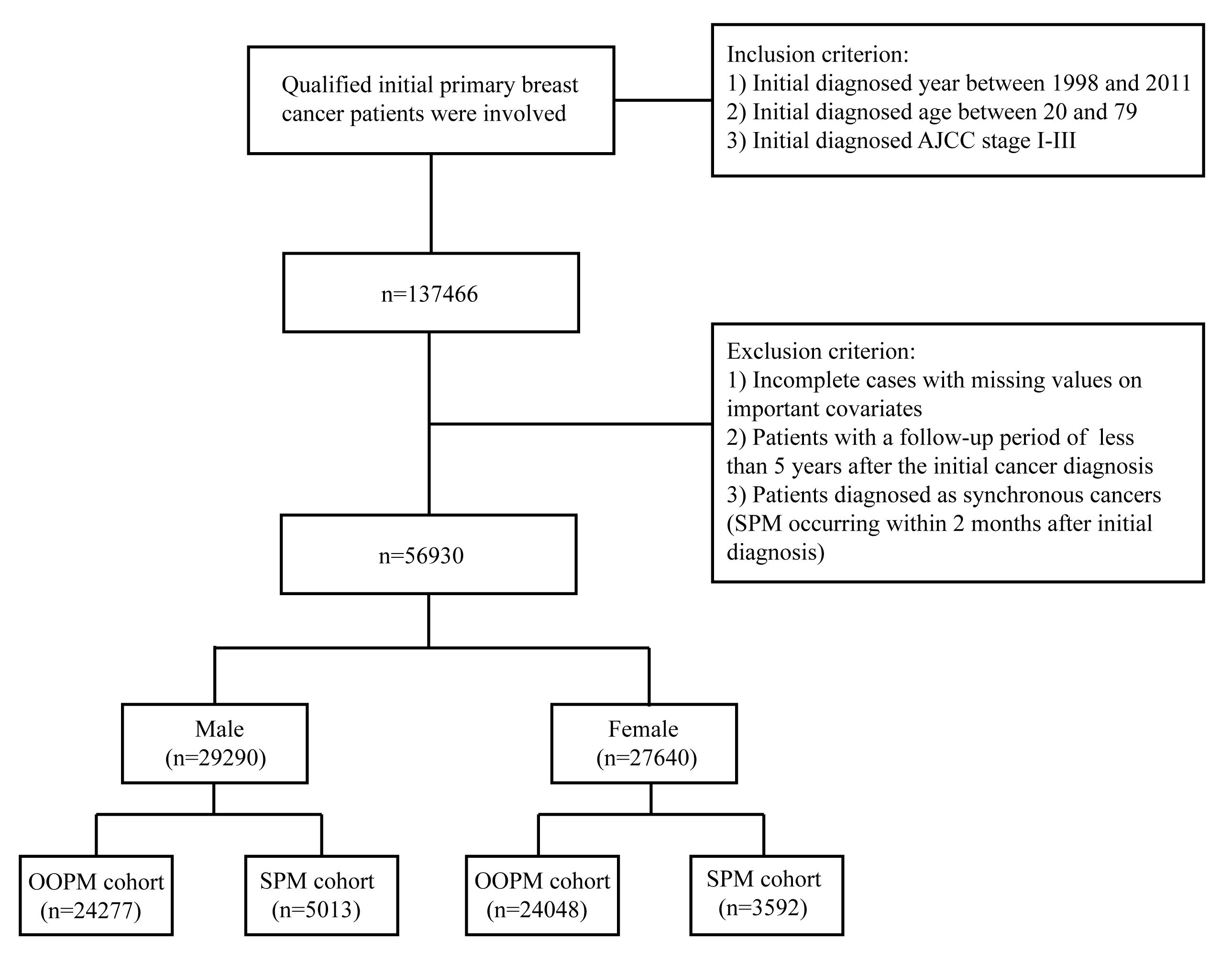

Supplement: Supplementary Figure 1 — The flowchart of cases selection. [file Image_1.TIF]

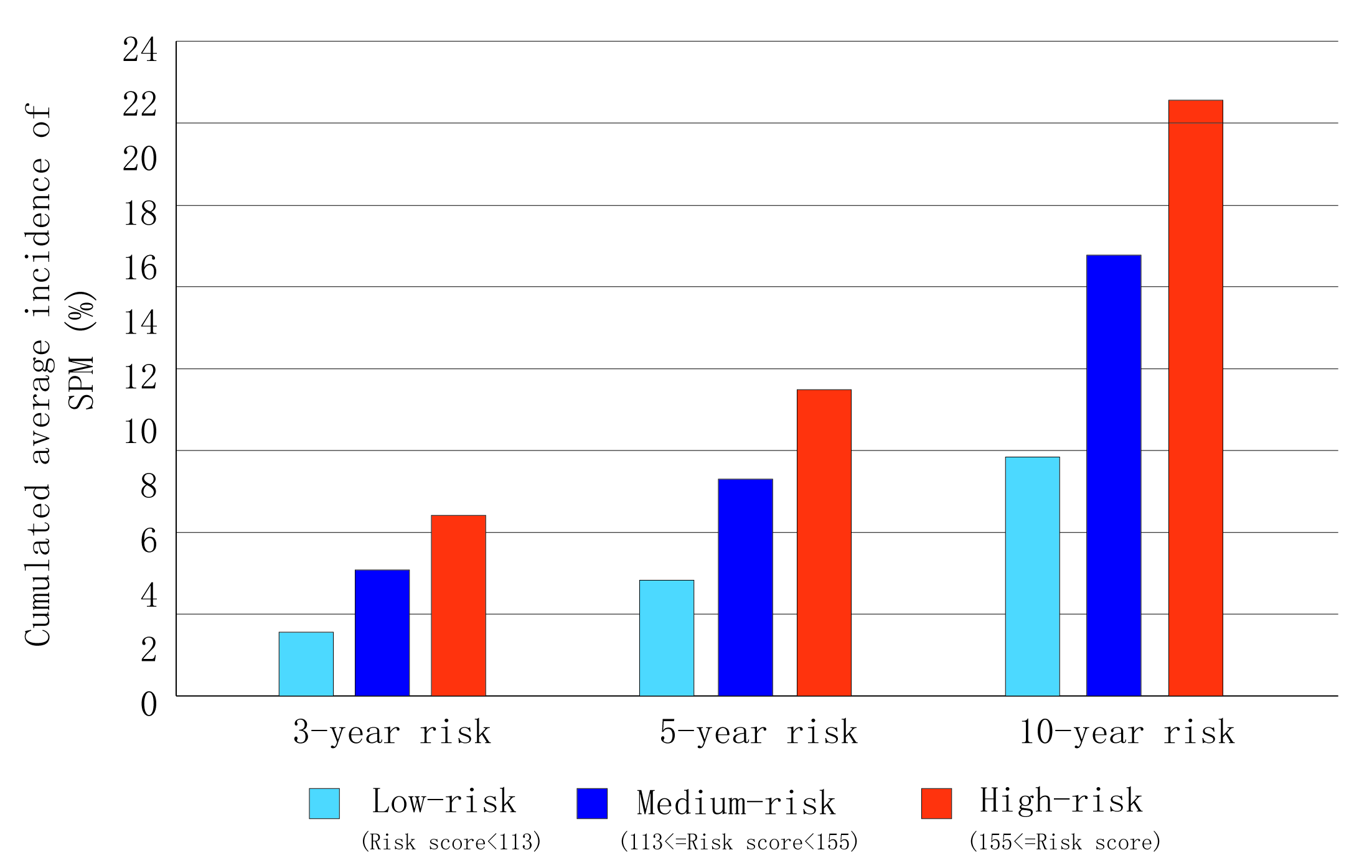

Supplement: Supplementary Figure 2 — The 3-, 5-, and 10-year cumulative incidence rates of SPMs among low-, medium-, and high-risk groups of male survivors. Risk stratification was conducted by the 25th and 75th percentile values of the risk score in the nomogram, which is 113 and 155, respectively. The cumulated incidence rates of these groups were assessed using the Fine-Gray method. Compared with the low-risk group (light-blue bar), the high-risk group (orange bar) exhibited a significantly higher cumulated incidence in 3-, 5-, and 10-year after the initial diagnosis. [file Image_2.TIF]

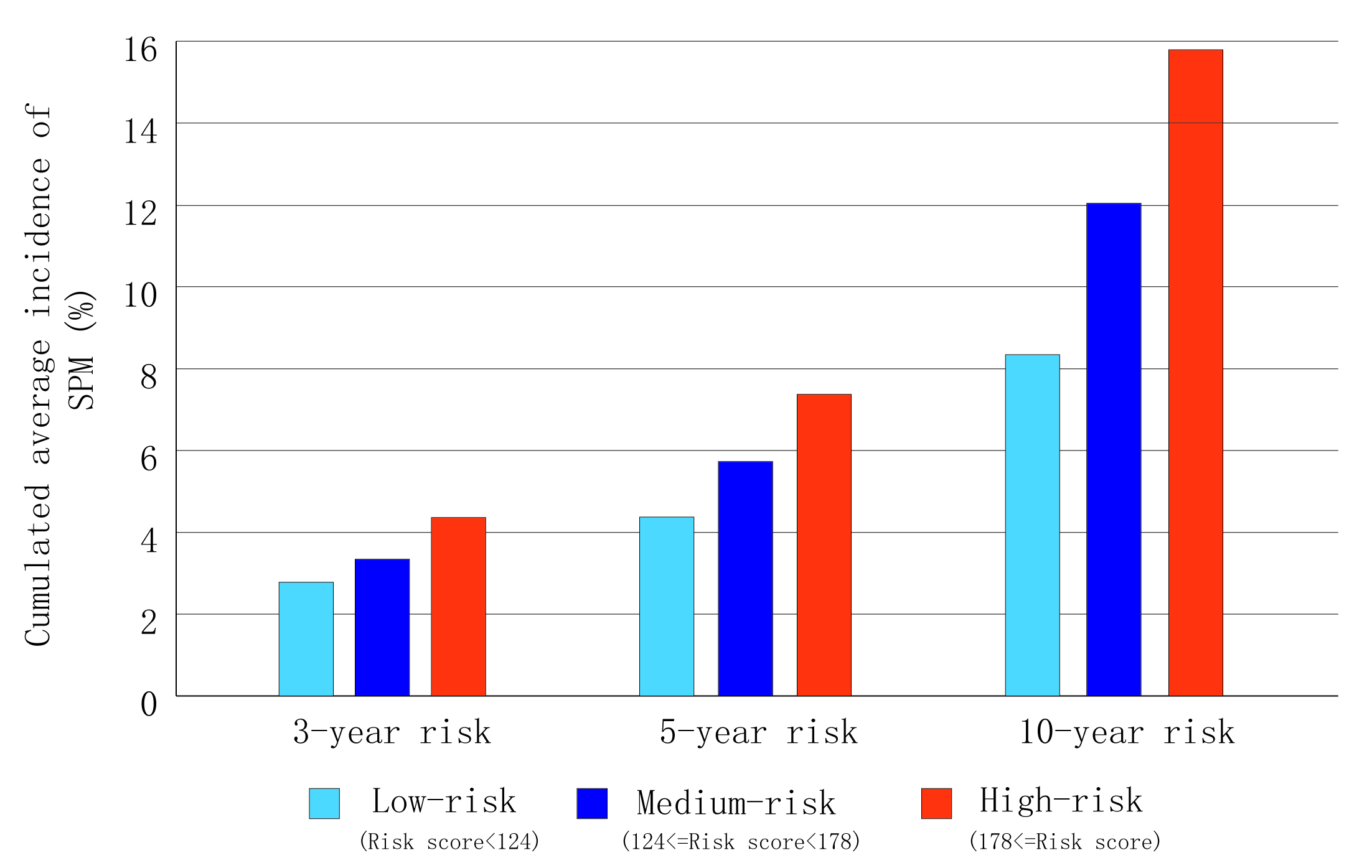

Supplement: Supplementary Figure 3 — The 3-, 5-, and 10-year cumulative incidence rates of SPMs among low-, medium-, and high-risk groups of female survivors. Risk stratification was conducted by the 25th and 75th percentile values of the risk score in the nomogram, which is 124 and 178, respectively. The cumulated incidence rates of these groups were assessed using the Fine-Gray method. Compared with the low-risk group (light-blue bar), the high-risk group (orange bar) exhibited a significantly higher cumulated incidence in 3-, 5-, and 10-year after the initial diagnosis. [file Image_3.TIF]

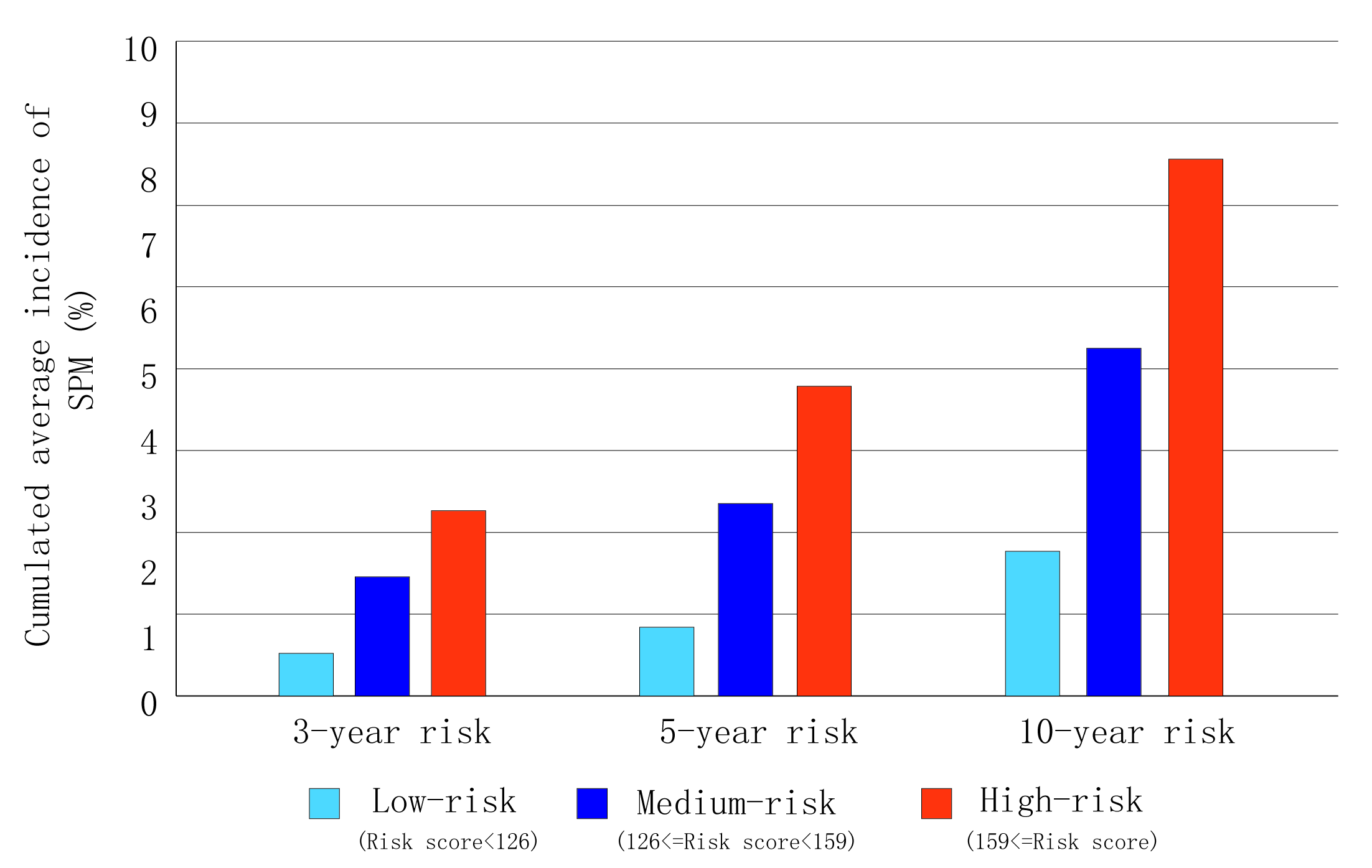

Supplement: Supplementary Figure 4 — The 3-, 5-, and 10-year cumulative incidence rates of second prostate carcinoma among low-, medium-, and high-risk groups of male survivors. Risk stratification was conducted by the 25th and 75th percentile values of the risk score in the nomogram, which is 126 and 159, respectively. The cumulated incidence rates of these groups were assessed using the Fine-Gray method. Compared with the low-risk group (light-blue bar), the high-risk group (orange bar) exhibited a significantly higher cumulated incidence in 3-, 5-, and 10-year after the initial diagnosis. [file Image_4.TIF]

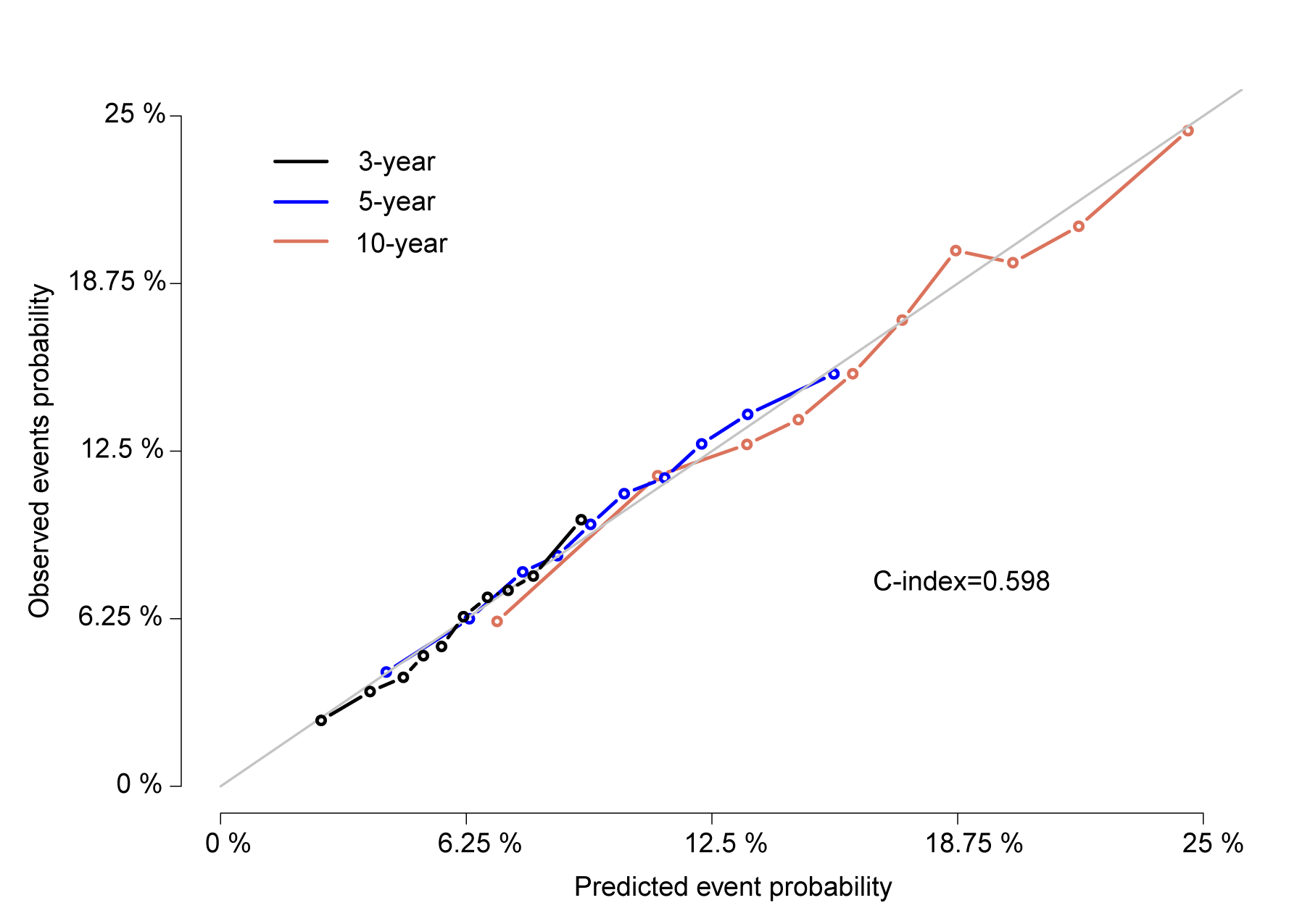

Supplement: Supplementary Figure 5 — Calibration curves for 3-year prediction (black line), 5-year prediction (blue line) and 10-year prediction (orange line) in male survivors. X-axis: predicted probability of developing second primary malignancy based on the multivariate competing risk model. Y-axis: Observed cumulative probability. [file Image_5.TIF]

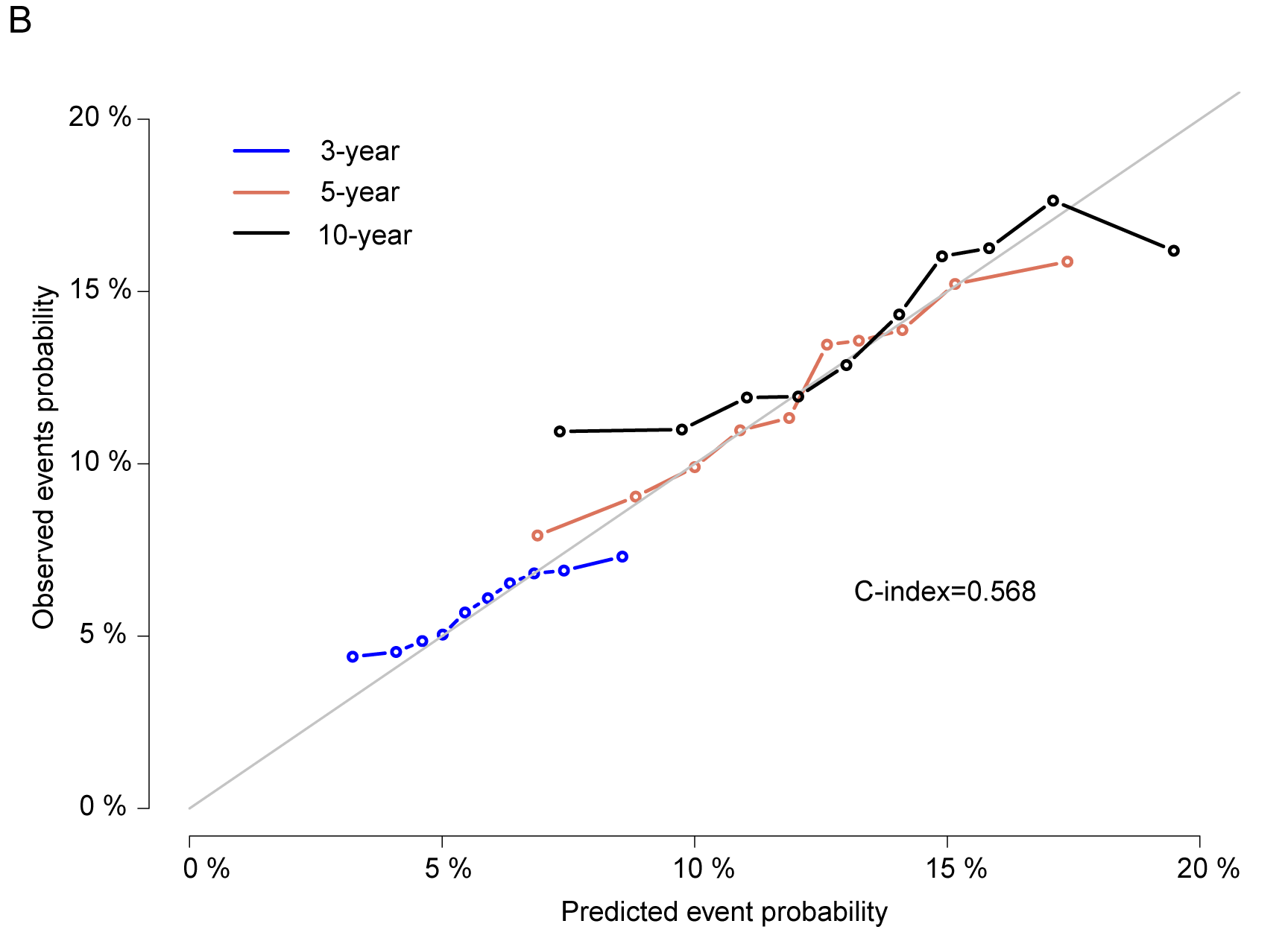

Supplement: Supplementary Figure 6 — Calibration curves for 3-year prediction (black line), 5-year prediction (blue line), and 10-year prediction (orange line) in female survivors. X-axis: predicted probability of developing second primary malignancy based on the multivariate competing risk model. Y-axis: Observed cumulative probability. [file Image_6.TIF]

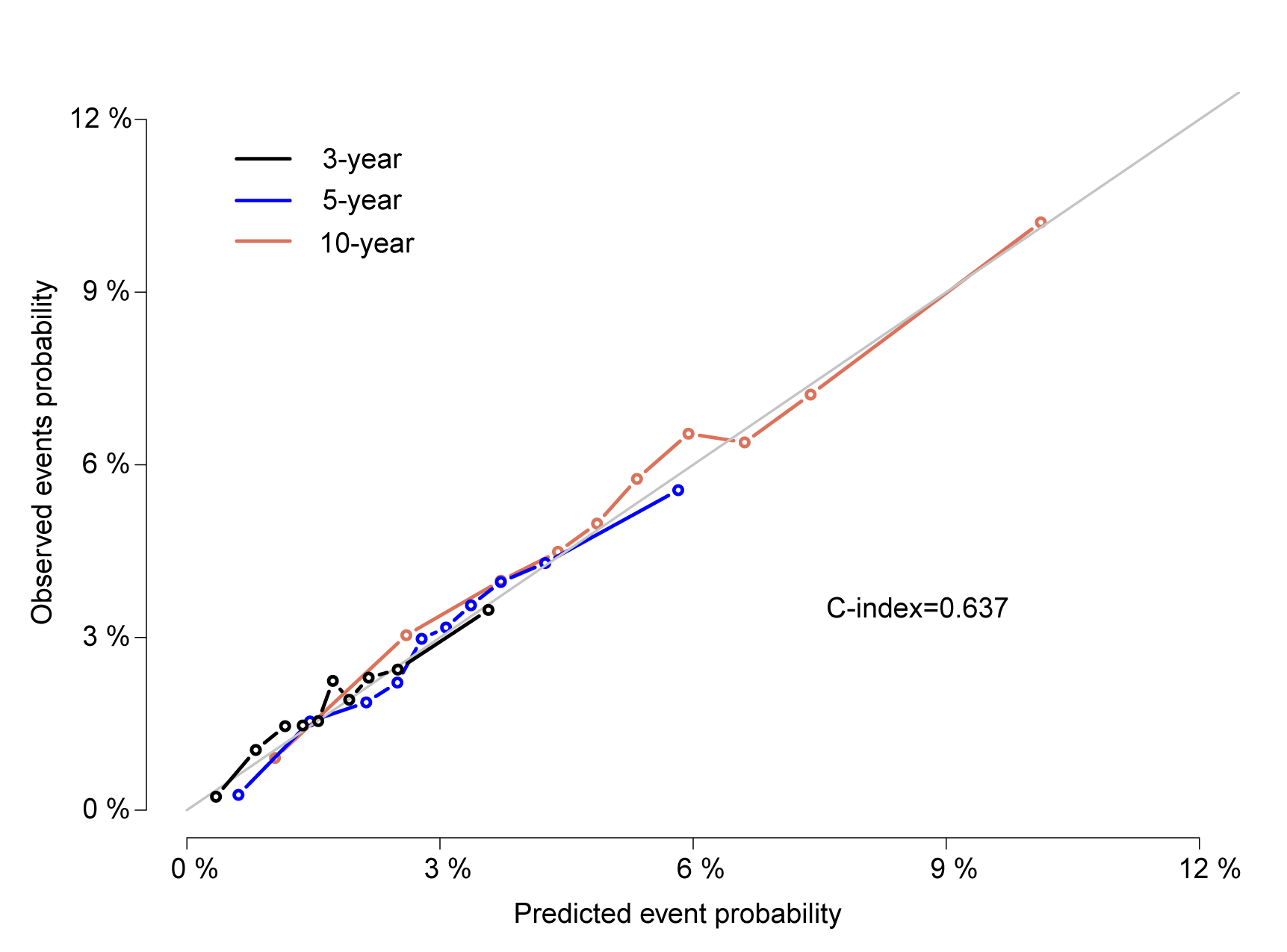

Supplement: Supplementary Figure 7 — Calibration curves for 3-year prediction (black line), 5-year prediction (blue line), and 10-year prediction (orange line) in male survivors. X-axis: predicted probability of developing second prostate carcinoma based on the multivariate competing risk model. Y-axis: Observed cumulative probability. [file Image_7.TIF]
