# Supplementary material for: Risk of Second Primary Malignancies in Colon Cancer Patients Treated With Colectomy
Source: Front Oncol. 2020 Jul 16;10:1154. doi: 10.3389/fonc.2020.01154 (PMC7378742; doi:10.3389/fonc.2020.01154)
Supplement: Supplementary Table 1 — Point assignment and risk score in the nomograms. [file Table_1.DOCX]

**Supplement table 1 Point assignment and risk score in the nomograms.**

| **Variable** | **Score** | | |
| --- | --- | --- | --- |
|  | **Male with SPM** | **Female with SPM** | **Male with second prostate cancer** |
| **Age at initial diagnosis, years** |  |  |  |
| 18-49 | 0 | 0 | 0 |
| 50–64 | 65 | 56 | 81 |
| 65–79 | 100 | 100 | 100 |
| **Race** |  |  |  |
| White | 22 | 34 | 27 |
| Black | 40 | 32 | 68 |
| Other | 0 | 0 | 0 |
| **Marital status** |  |  |  |
| Married | 6 |  | 14 |
| Unmarried | 0 |  | 0 |
| **Initial diagnosed site** |  |  |  |
| Right colon |  | 10 | 10 |
| Left colon |  | 0 | 0 |
| **Tumor size, cm** |  |  |  |
| <3 | 0 | 0 | 0 |
| 3-5 | 17 | 10 | 8 |
| >5 | 34 | 20 | 17 |
| **Lymph nodes examined** |  |  |  |
| <12 | 10 |  | 11 |
| >=12 | 0 |  | 0 |
| **Tumor grade** |  |  |  |
| I-II |  | 0 |  |
| III-IV |  | 21 |  |
| **Stage** |  |  |  |
| I | 20 | 30 |  |
| II | 10 | 30 |  |
| III | 0 | 0 |  |
| **Chemotherapy** |  |  |  |
| No/Unknown |  | 0 |  |
| Yes |  | 24 |  |
